# Nutritional consulting in regular veterinary practices in Belgium and the Netherlands

**DOI:** 10.1002/vms3.679

**Published:** 2021-11-29

**Authors:** Niels R. Blees, Veerle L. Vandendriessche, Ronald J. Corbee, Philippe Picavet, Myriam Hesta

**Affiliations:** ^1^ Department of Clinical Sciences of Companion Animals Faculty of Veterinary Medicine Utrecht University Utrecht The Netherlands; ^2^ Pavo Heijen The Netherlands; ^3^ Hill's Pet Nutrition Brussels Belgium; ^4^ Department of Nutrition Genetics and Ethology Ghent University Merelbeke Belgium

**Keywords:** body condition score, diet, muscle condition score, nutritional assessment

## Abstract

**Background:**

Increased interest in nutrition by dog and cat owners stresses the importance of providing tailored nutritional guidance for each patient by veterinarians. The World Small Animal Veterinary Association (WSAVA) has provided guidelines to help veterinarians implement this in every‐day patient care, by screening patients for the presence of nutritional risk factors, establishing tailored nutritional plans and providing adequate patient follow‐up tools.

**Objectives:**

This study aimed to assess the use of nutritional assessments in companion animal practices, and to investigate differences between Dutch and Belgian veterinarians.

**Methods:**

A survey was conducted among Dutch and Belgian veterinarians. Of the 423 respondents, 53% were from Belgium, and 47% were from the Netherlands.

**Results:**

Only 21% had prior knowledge of the WSAVA nutritional assessment guidelines. General trends in the usage of nutritional assessments were similar in the examined countries. Aside from weighing, diet evaluation by collecting dietary information and body condition or muscle condition scoring were used infrequently, mostly due to insufficient knowledge of the methods. Nutritional recommendations were often made as part of a treatment plan, and were mostly made by veterinarians, but in Dutch practices also by veterinary nurses.

**Conclusion:**

Despite the fact that nutritional recommendations are a regular part of treatment plans, nutritional risk factors may be missed due to a lack of completely performed nutritional assessments. It remains important to promote the benefits of regular nutritional assessments to veterinarians, which will improve patients’ health.

## Graphical abstract

This study aimed to assess the use of complete nutritional consultation in regular veterinary care. The results of this study show that implementation of nutrition in veterinary care is generally low, and efforts should be made to raise awareness on the benefits of regular nutritional care.

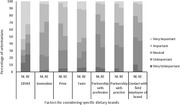

## 1 INTRODUCTION

Nutrition is an important topic in veterinary practice (Bergler et al., 2016; Vandendriessche et al., 2017). Increased awareness of nutrition‐related diseases and popularity of alternative diets have made veterinarians indispensable for early detection of nutritional imbalances and risk factors (Dillitzer et al., 2011; Laflamme et al., 2008; Rajagopaul et al., 2016). Hence, it is essential for veterinarians to provide tailored nutritional guidance for each patient at every consultation to provide optimal patient care (Freeman et al., 2011).

To aid veterinarians in incorporating complete nutritional assessments in regular patient care, global nutritional assessment guidelines were developed by the World Small Animal Veterinary Association (WSAVA) (Freeman et al., 2011). These guidelines describe inclusion of nutritional assessment as the fifth vital assessment in regular patient care. This starts with identification of nutritional risk factors for disease through a complete dietary and lifestyle history, and bodyweight and body composition assessment through body condition and muscle condition scoring. Evaluation of these factors results in tailored nutritional recommendations, a feeding management strategy, and a follow‐up plan with regular monitoring and compliance assessment.

Despite these efforts, routine implementation of nutritional consultations and assessments remains limited, and dietary recommendations are often provided after development of nutrition‐related diseases (Bergler et al., 2016; MacMartin et al., 2015; Rolph et al., 2014; Siebert et al., 2016). For instance, routine assessment of body composition is uncommon; therefore, leaving development of sarcopenia or obesity to go unnoticed, impairing nutrition's preventive role (Bruckner & Handl, 2020; Freeman, 2012).

Most of the aforementioned studies describe specific parts of nutritional assessment, and vary in study design. For instance, some included estimates of veterinarians or pet owners (Bergler et al., 2016; Siebert et al., 2016; Vandendriessche et al., 2017), while others included objective conversation analysis of consultations (MacMartin et al., 2015) or retrospective analyses of patient files (German & Morgan, 2008; Rolph et al., 2014). These differences make them difficult to compare and draw conclusions on the integration of nutritional assessment in clinical practice. Additionally, the diversity in geographical locations could limit extrapolation of the results to other countries, as veterinarians in different regions are expected to have different attitudes and education in small animal nutrition (Becvarova et al., 2016). Thus, this study was conducted to assess the incorporation of the methods as described by the WSAVA nutritional assessment guidelines (Freeman et al., 2011) in veterinary practices in Belgium and the Netherlands. Furthermore, differences between both countries regarding nutritional assessments were studied.

## 2 MATERIALS AND METHODS

### 2.1 Data collection

An anonymous web‐based questionnaire (www.surveymonkey.com) was distributed to Belgian and Dutch veterinarians via dedicated social media groups and the newsletter of Utrecht University, the Netherlands. As the WSAVA nutritional assessment guidelines are mostly aimed towards dogs and cats veterinarians that treat animal species other than dogs and cats as the majority of their patients were excluded from this study. The survey was accessible between May and June 2016 in Belgium and between February and May 2018 in the Netherlands. Complete sections of incomplete questionnaires were included in the analysis.

### 2.2 Survey

The survey consisted of three main sections, with a total of 66 multiple choice and open‐ended questions. The first section of the survey consisted of 17 questions about practice demographics (Appendix). Estimates of the proportion of cats, dogs, and exotic animal species as percentage of the responding veterinarians’ total number of patients on a yearly basis were obtained. These questions were followed by inquires on the number of staff and their function in the respondent's practice, their years of experience since graduation, and whether any members of the veterinary health care team were specifically responsible for nutritional consultation. If applicable, the educational background of this team member was asked. Lastly, participation in nutrition‐focused continuing education was explored.

The second section consisted of 25 questions about nutritional screening. The veterinarians were asked to provide estimates on the frequency in which they measured and registered weight, body condition score (BCS) and muscle condition score (MCS) of their patients, the specifications on the used scoring system, and to specify reasons for infrequent use of these methods. Next, estimates on the use of specific dietary history questions, such as the type of food that is being fed to the patient and the food amount, and the use of nutritional surveys were obtained. The respondents were also asked whether they were familiar with the WSAVA nutritional assessment guidelines.

The third section contained 24 questions which concerned the role of nutrition in the respondents’ practices. Information was obtained on the sale of maintenance and therapeutic or raw food diets in the respondents’ practices. The respondents were then asked about their criteria for recommending a specific brand of food. The estimated frequency in which dietary change is recommended for life stages and as component of the management of diseases, as well as the use of homemade diets as part of a treatment were obtained. The next question gave information on the members of the veterinary health care team that recommended dietary change. Next, estimates of assumed owner compliance to nutritional recommendations were asked, as well as suggested reasons for non‐compliance. These were followed by inquiries about the means of follow‐up which the respondents used, and their methods to increase compliance with owners. Lastly, the respondents were asked for specific occasions in which owners were informed of nutritional necessities, such as obesity clinics and senior consultations, and whether the practice organised educational meetings for owners.

### 2.3 Statistical analyses

Data were exported to Microsoft Excel 2016 (version 1808, Microsoft Redmond, WA, USA) and statistical analyses were performed using IBM SPSS Statistics (version 25.0. IBM Corporation, Armonk, NY, USA). Numerical and categorical data were displayed as median (interquartile range) and percentage (number of observations), respectively. Due to the discrete nature of the data, differences between Belgian and Dutch respondents were assessed with Mann–Whitney *U* tests for numerical data and Chi‐square analyses with continuity corrections for categorical data. Cramér's V was calculated to assess the effect size of significant and trend results (Kim, 2017). Statistical significance was set at *p* < 0.05 and trend results as 0.05 < *p* < 0.10. Data were presented per country when a significant or trend difference between countries was found.

## 3 RESULTS

### 3.1 Respondent participation

A total of 423 questionnaires were submitted, 52.5% (222/423) from Belgian veterinarians and 47.5% (201/423) from Dutch veterinarians. Ten respondents indicated treating species other than dogs and cats as the majority of their patients and were excluded from further analysis. A total of 60.9% (252/413) veterinarians completed all questions of the survey, 66.3% (167/252) from Belgium and 33.7% (85/252) from the Netherlands.

### 3.2 Respondent population

An overview of the participating respondents’ practices is presented in Table 1. Most responding veterinarians worked in practices with more than one veterinarian, and only 49.8% (110/221) of the Belgian and 15.9% (21/132) of the Dutch respondents worked in ‘single‐vet’ practices (*p* < 0.001, *φ* = 0.339). A larger proportion of Belgian veterinary practitioners had more years of experience since graduation. The majority of veterinary employees in practices were female, but the proportion of male veterinarians was higher in Belgian practices compared to Dutch practices. Veterinary nurses were often available in Dutch practices, but were not common in Belgian practices.

**TABLE 1:** An overview of the practice demographics of the survey respondents and comparisons between Belgian and Dutch practices, displayed as number of employees or as percentages of the employees

		NL			BE			
Practice information		*n*	Median	IQR	*n*	Median	IQR	*p*
Total number of veterinarians		132	3	2–4	221	2	1–2	<0.001^a^
Number of veterinary nurses		133	4	3–6	221	0	0–1	<0.001^a^
Employment per proportion of veterinarians	Full‐time	133	33%	0%–61%	221	100%	67%–100%	<0.001^a^
	Part‐time		67%	39%–100%		0%	0%–33%	<0.001^a^
Proportion of veterinarians of each gender	Female	132	80%	50%–100%	220	67%	27%–100%	0.006^a^
	Male		20%	0%–40 %		33%	0%–66%	0.014^a^
Attendance of continuous education lectures in nutrition	Veterinarian	129	20%	0%–50%	222	100%	54%–100%	<0.001^a^
	Veterinary technician		50%	0%–80%		0%	0%–0%	<0.001^a^
Years of experience as proportion of veterinarians	0–5 years	132	14%	0%–38%	221	0%	0%–25%	<0.001^a^
	5–10 years		0%	0%–33%		0%	0%–0%	0.001^a^
	10–20 years		27%	0%–50%		0%	0%–50%	0.078^b^
	>20 years		0%	0%–50%		33%	0%–100%	0.004^a^
Proportion of nutritional recommendations made by	Veterinarian	98	80%	50%–85%	178	100%	80%–100%	<0.001^a^
	Veterinary technician		20%	15%–50%		0%	0%–20%	<0.001^a^

Abbreviations: BE, Belgian respondents; IQR, interquartile range; NL, Dutch respondents.

^a^ Significant difference between both countries by Mann–Whitney *U* test (*p* < 0.05).

^b^ Trend difference between countries by Mann–Whitney *U* test (0.10 > *p* > 0.05).

**FIGURE 1:**
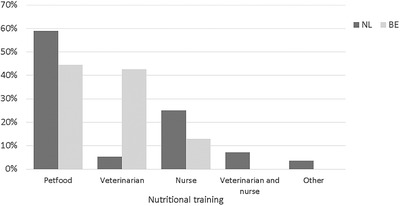
An overview of the training of the employee that is responsible for nutritional consulting in the respondents’ practices. ‘Petfood’ indicates an unspecified employee that is trained by a petfood company, ‘other’ includes non‐veterinary personnel

Less than half of Belgian [23.4% (52/222)] and Dutch [45.0% (58/129)] practices had dedicated employees for nutritional consultation (*p* < 0.001, *φ* = 0.224), which included team members trained by pet food companies (Figure 1). Continuing education in nutrition was attended by members of most practices [Belgian respondents (BE): 59.9% (133/222), Dutch respondents (NL): 80.6% (104/129), *p* < 0.001, *φ* = 0.213]. In Belgian practices, continuing education in nutrition was mostly attended by veterinarians, while veterinary nurses attended these courses more often in Dutch practices.

### 3.3 Nutritional evaluation

Only 21.3% (67/314) of the respondents had prior knowledge of the WSAVA nutritional guidelines (*p* = 0.783). The proportion of consultations in which a nutritional evaluation was performed and documented is summarised in Tables 2 and 3. Belgian veterinarians tended to inquire about diet and feeding habits more often, while Dutch respondents were more likely to add nutritional information to the patient's record. Only 7.9% (25/315, *p* = 0.786) used dietary questionnaires.

**TABLE 2:** Overview of the estimated percentages of consultations in which the respondents perform nutritional evaluations, and comparisons between Belgian and Dutch respondents

		Frequency of use during consultations						
		NL			BE			
Nutritional evaluations		*n*	Median (%)	IQR (%)	*n*	Median (%)	IQR (%)	*p*
Nutritional examinations	Weighing	127	95	90–99	217	95	80–100	0.5
	BCS	127	20	0–70	217	20	0–50	0.515
	MCS	124	0	0–0	210	0	0–2	0.095^b^
Diet history	Type of diet	115	50	25–75	199	75	50–90	<0.001^a^
	Amount of food	115	40	20–60	199	50	25–80	<0.001^a^
	Number of meals	115	30	10–60	199	50	25–80	<0.001^a^
	Number of treats	115	30	20–65	199	50	25–80	<0.001^a^
	Housing environments	115	20	5–50	199	40	15–80	0.82
	Activity	115	40	10–75	199	50	20–80	0.037^a^

Abbreviations: BE, Belgian respondents; BCS, body condition score; IQR, interquartile range; MCS, muscle condition score; NL, Dutch respondents.

^a^ Significant difference between countries by Mann–Whitney *U* test (*p* < 0.05).

^b^ Trend difference between countries by Mann–Whitney *U* test (0.10 > *p* > 0.05).

**TABLE 3:** Overview of the estimated percentages of consultations in which the respondents document nutritional evaluation methods in the patient's record after assessment, and comparisons between Belgian and Dutch respondents

		Frequency of documentation in patient's record when used						
		NL			BE			
Nutritional evaluations		*n*	Median (%)	IQR (%)	*n*	Median (%)	IQR (%)	*p*
Nutritional examinations	Weighing	127	95	90–100	217	95	78–100	0.08^b^
	BCS	127	20	0–65	217	5	0–40	0.001^a^
	MCS	124	0	0–0	210	0	0–0	0.043^a^
Diet history	Type of diet	115	60	20–100	199	25	0–80	<0.001^a^
	Amount of food	115	50	10–80	199	10	0–50	<0.001^a^
	Number of meals	115	50	10–80	199	10	0–50	<0.001^a^
	Number of treats	115	40	5–80	199	10	0–50	<0.001^a^
	Housing environments	115	30	0–80	199	1	0–50	<0.001^a^
	Activity	115	40	5–80	199	10	0–50	<0.001^a^

Abbreviations: BE, Belgian respondents; BCS, body condition score; IQR, interquartile range; MCS, muscle condition score; NL, Dutch respondents.

^a^ Significant difference between countries by Mann–Whitney *U* test (*p* < 0.05).

^b^ Trend difference between countries by Mann–Whitney *U* test (0.10 > *p* > 0.05).

All respondents (*n* = 351) had at least one scale in their practice, and 38.3% (85/222) of Belgian and 86.0% (111/129) of Dutch respondents had separate scales for weighing cats (*p* < 0.001, *φ* = 0.464). Observed changes in weight were discussed with owners by almost all respondents [99.4% (342/344), *p* = 0.726]. Additionally, 70.3% (242/344, *p* = 0.837) used BCS in their consultations, albeit infrequently. Most Belgian respondents used a five‐point scale [81.6% (124/152)], whereas most Dutch respondents used a nine‐point scale [69.0% (60/87), *p* < 0.001, *φ* = 0.504]. A minority of the respondents used MCS during consultation [23.7% (79/334), *p* = 0.120]. Reasons for non‐usage are listed in Table 4.

**TABLE 4:** Volunteered reasons for irregular use of body condition scoring and muscle condition scoring, given by responding veterinarians (*n* = 276)

	BCS		MCS	
Reason	Number of observations	Percentage	Number of observations	Percentage
Insufficient experience or habit in performing the method	71	27.2%	0	0.0%
Insufficient knowledge of the performance or use of the method	21	8.0%	141	60.0%
Time constraints during consultation	59	22.6%	20	8.5%
Use only when relevant to clinical signs	53	20.3%	30	12.8%
Methods are not in the interest of the owner and the animal	28	10.7%	29	12.3%
Use of other nutritional assessment is enough	29	11.1%	15	6.4%

Abbreviations: BCS, body condition score; MCS, muscle condition score.

### 3.4 Nutritional sales and recommendations

**FIGURE 2:**
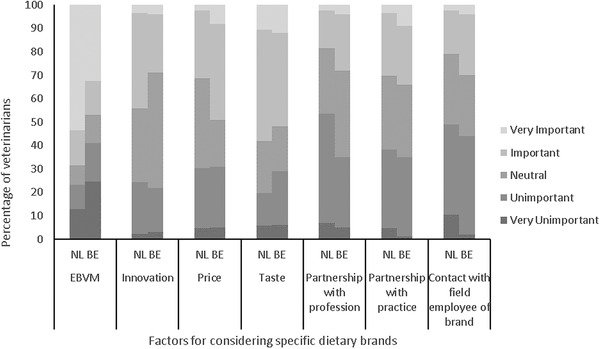
The factors that influence the participating veterinarians in the selection of specific dietary brands or types. Abbreviations: BE, Belgian veterinarians; EBVM, Evidence‐based veterinary medicine; NL, Dutch veterinarians

All respondents (*n* = 293) sold pet food at their practice. Therapeutic diets were sold most [99.7% (292/293), *p* = 1.000], followed by maintenance diets [87.7% (257/293), *p* = 0.761] and raw‐meat diets [5.0% (21/293), *p* = 0.652]. Practices sold approximately three (2–4) different brands of diets (*p* = 0.155). Evidence based veterinary medicine was considered the most important factor for dietary recommendations (Figure 2).

Dietary recommendations were often made for cats diagnosed with renal disease, lower urinary tract disease, or diabetes mellitus (Table 5), whereas dogs obtained most recommendations for renal and orthopaedic disease, and overweight/obesity (Table 6). Homemade diets were recommended by 75.7% (193/255) respondents (*p* = 0.11), but their recipes often lacked sources of essential fatty acids [BE: 80.0% (80.0%–100%), NL: 0.0% (0.0%–50.0%), *p* < 0.001] and essential vitamins [BE: 50.0% (0.0%–100%), NL: 0.0% (0.0%–7.5%)]. Knowledge about the composition of home‐cooked diets (HCD) originated often from self‐acquired knowledge [BE: 90.3% (131/145), NL: 73.9% (51/69)] rather than from board‐certified diplomates in nutrition [BE: 9.7% ( 14/145), NL: 26.1% (18/69), *p* = 0.003, *φ* = 0.215], which were present in both countries at the time of the survey.

**TABLE 5:** The estimated percentage of cats with specific indications in which dietary recommendations are given and difference between Belgian and Dutch respondents (NL: N = 105; BE: N = 189)

		NL		BE		
Indication		Median (%)	IQR (%)	Median (%)	IQR (%)	*p*
Disease	Lower urinary tract disease	100	88–100	100	93–100	0.048^a^
	Overweight	80	50–100	80	53–100	0.085^b^
	Diabetes mellitus	100	78–100	100	65–100	0.702
	Orthopaedic disease	75	50–90	50	20–80	0.004^a^
	Renal disease	100	100–100	100	100–100	0.023^a^
	Gastrointestinal disease	75	50–100	80	50–100	0.930
	Dermatologic disease	75	50–100	50	30–83	0.001^a^
	Liver disease	50	30–83	70	25–100	0.028^a^
	Cardiovascular disease	0	0–20	20	0–50	0.001^a^
	Dental disease	50	25–80	30	5–60	<0.001^a^
	Hyperthyroidism	25	5–50	40	0–80	0.833
Life stage	Growth	80	20–100	80	40–100	0.147
	Adult	20	10–63	50	20–100	0.001^a^
	Senior	50	20–80	70	50–100	0.004^a^
	Neuter	75	28–100	80	50–100	0.091^b^

Abbreviations: BE, Belgian respondents; IQR, interquartile range; NL, Dutch respondents.

^a^ Significant difference between countries by Mann–Whitney *U* test (*p* < 0.05).

^b^ Trend difference between countries by Mann–Whitney *U* test (0.10 > p > 0.05).

**TABLE 6:** The estimated percentage of dogs with specific indications in which dietary recommendations are given and difference between Belgian and Dutch respondents (NL: N = 98; BE: N = 177)

		NL		BE		
Indication		Median (%)	IQR (%)	Median (%)	IQR (%)	*p*
Disease	Lower urinary tract disease	70	48–100	100	70–100	<0.001^a^
	Overweight	80	50–100	90	60–100	0.001^a^
	Diabetes mellitus	75	20–100	100	50–100	<0.001^a^
	Orthopaedic disease	80	50–96	75	50–98	0.301
	Renal disease	100	100–100	100	90–100	0.271
	Gastrointestinal disease	80	50–100	75	50–100	0.425
	Dermatologic disease	80	68–100	80	50–100	0.027^a^
	Liver disease	50	20–93	75	50–100	0.003^a^
	Cardiovascular disease	4	0–30	25	0–63	<0.001^a^
	Dental disease	50	20–80	50	10–70	0.04^a^
Life stage	Growth	80	45–100	100	50–100	0.274
	Adult	50	25–100	50	20–80	<0.001^a^
	Senior	50	20–80	70	45–100	<0.001^a^
	Neuter	50	19–96	80	30–100	<0.001^a^

Abbreviations: BE, Belgian respondents; IQR, interquartile range; NL, Dutch respondents.

^a^ Significant difference between countries by Mann–Whitney *U* test (*p* < 0.05).

Approximately half of the clients were estimated to initiate feeding according to the prescribed feeding plan, and approximately half of these clients completed the dietary regimen. Feeding costs were considered the main reason for non‐compliance (Table 7). The respondents also suggested difficulty to maintain a strict diet, cheaper and more easily acquirable other foods, conflicting nutritional recommendations from others such as a breeder or friend, or a lack of long‐term motivation, as common reasons for non‐compliance.

**TABLE 7:** The percentage of owners that complies and adheres to nutritional recommendations as estimated by the responding veterinarians, and the frequency of commonly heard reasons of non‐adherence with differences between Belgian and Dutch respondents (NL: N = 86; BE: N = 171)

		NL		BE		
Compliance and adherence		Median (%)	IQR (%)	Median (%)	IQR (%)	*p*
Percentage of owners who comply with nutritional advice		50	30–60	50	35–75	0.117
Percentage of owners who complete the prescribed dietary regimen		50	19–70	50	30–75	0.001^a^
Percentage of reasons of owners who do not adhere to nutritional advice	Prescribed diet is too expensive	50	29–75	50	35–80	0.115
	Prescribed diet is not tasteful	10	5–30	20	10–40	0.001^a^
	No improvement on recommended diet	10	5–25	20	10–30	0.058^b^
	Owner's opinion that animal has improved enough	10	5–30	20	5–40	0.389
	Other	0	0–10	0	0–5	0.010^a^

Abbreviations: BE, Belgian respondents; IQR, interquartile range; NL, Dutch respondents.

^a^ Significant difference between countries by Mann–Whitney *U* test (*p* < 0.05).

^b^ Trend difference between countries by Mann–Whitney *U* test (0.10 > *p* > 0.05).

The respondents commonly indicated differences in the amount of food that had to be fed, for example the ability to feed more kibble in weight loss diets while retaining the same caloric intake, and recommended gradual transition to the new diet to increase compliance (Table 8). Nutritional recommendations were commonly monitored by follow‐up consultations and telephone calls (Table 9).

**TABLE 8:** An overview on the methods of monitoring that are being used by the respondents to monitor the effects of dietary recommendations and difference between Belgian and Dutch respondents, with association with the factor ‘country’ (NL: N = 79; BE: N = 170)

	NL		BE			
Follow‐up method	Number of veterinarians	Percentage	Number of veterinarians	Percentage	*p*	Cramér's V
Subsequent consultation	71	89.9%	116	68.2%	<0.001^a^	0.233
Contact by telephone	75	94.9%	91	53.5%	<0.001^a^	0.409
Written communication	34	43.0%	31	18.2%	<0.001^a^	0.263

Abbreviations: BE, Belgian respondents; NL, Dutch respondents.

^a^ Significant difference between countries by chi‐squared test (*p* < 0.05).

**TABLE 9:** An overview of the different methods that are used by the respondents to increase compliance of dietary recommendations and a comparison between Belgian and Dutch respondents, and the association with factor ‘country’ (NL: N = 86; BE: N = 171)

	NL		BE			
Method of motivation	Number of veterinarians	Percentage	Number of veterinarians	Percentage	*p*	Cramér's V
Indicating differences in food amount	76	88.4%	143	83.6%	0.312	0.063
Use of gradual transition	71	82.6%	129	77.8%	0.195	0.081
Difference in the price of the products	35	34.9%	77	45.0%	0.120	0.097
Calculations of the difference in price per kilogram food	14	16.3%	28	16.4%	0.984	0.001
Selling small sample packages^a^	55	64.0%	67	39.2%	<0.001^a^	0.234
Use of free sample packages^a^	31	36.0%	118	69.0%	<0.001^a^	0.315

Abbreviations: BE, Belgian respondents; NL, Dutch respondents.

^a^ Significant difference between countries by chi‐squared test (*p *< 0.05).

### 3.5 Client education

Many respondents educated their clients on the risks of becoming overweight after neutering and recommended adjustments to the animal's diet, whereas growth‐related nutritional risks were discussed less often (Table 10). Some respondents indicated organizing specific opportunities to educate clients, such as obesity clinics and senior consultations [BE: 46/122 (37.7%), NL: 46/85 (54.1%), *p* < 0.001, *φ* = 0.263].

**TABLE 10:** The percentage of clients in which the respondents inform of specific nutrition‐related risks after neutering and during growth and comparisons between Belgian and Dutch respondents (NL: N = 85; BE: N = 168)

		NL		BE		
Risk		Median (%)	IQR (%)	Median (%)	IQR (%)	*p*
Neutering	The risk that the animal becomes overweight	100	80–100	100	90–100	0.087^b^
	Lowering the amount of food or using a low‐calorie diet	100	80–100	100	90–100	0.067^b^
Growth	Necessity to use a diet specifically for a growing animal	90	70–100	100	80–100	<0.001^a^
	The time in which nutrition can be switched to an adult diet	60	50–85	90	70–100	<0.001^a^
	The risks of giving too many treats during growth	50	20–80	80	50–100	<0.001^a^
	The risks of giving extra supplements during growth	20	8–80	70	25–100	<0.001^a^

Abbreviations: BE, Belgian respondents; IQR, interquartile range; NL, Dutch respondents.

^a^ Significant difference between countries by Mann–Whitney *U* test (*p* < 0.05).

^b^ Trend difference between countries by Mann–Whitney *U* test (0.10 > *p* > 0.05).

## 4 DISCUSSION

Veterinarians play a key role in providing nutritional guidance for their patients, as the veterinary health care team is an important source of nutritional information for pet owners (AAHA, 2003; Schleicher et al., 2019). However, this study shows that nutritional assessment, as described by the WSAVA nutritional assessment guidelines (Freeman et al., 2011), is often omitted during consultation in veterinary practices in Belgium and the Netherlands. These trends tended to be similar in both countries, with differences mainly being present in practice demographics. Even though most data consisted of estimations by the responding veterinarians, the results give insights into the awareness and perceived use of nutritional assessments from the veterinarians’ point of view. Only 21% of the responding veterinarians had prior knowledge of the guidelines, demonstrating the need for more nutrition education and further promotion of these guidelines.

Tailored nutritional guidance can only be achieved by performing regular nutritional assessments (Freeman et al., 2011). For this purpose, a complete nutritional assessment encompasses thorough evaluations of dietary and lifestyle history, followed by patient bodyweight and body composition examinations, to identify nutritional risks. Only then, appropriate nutritional recommendations can be made. Yet, complete nutritional assessments were seldom performed by the respondents, even though nutritional recommendations were given in a variety of diseases. To illustrate, dietary information was collected infrequently, and questions were often limited to the animal's main source of food. Additionally, body composition assessment through BCS and MCS was infrequently performed by the respondents. These results match previous studies, which showed that complete nutritional assessments are seldom performed during consultation (Bergler et al., 2016; Bruckner & Handl, 2020; Lumbis & de Scally, 2020). As such, it has been suggested that veterinarians would assess nutrition only when it was relevant to the patients’ presenting complaint (Bruckner & Handl, 2020). The same situation applied to our respondents in the case of BCS assessment.

The irregularity in which complete nutritional assessments were performed might cause nutritional risk factors to go unnoticed, and has significant consequences for patient care (Bergler et al., 2016; Vandendriessche et al., 2017). For instance, failure to identify animals that are routinely fed unbalanced diets can lead to diet‐induced disorders, thus impeding patient health (Dillitzer et al., 2011). On the other hand, unnoticed dietary risk factors limit opportunities of early dietary intervention. In fact, animals in specific life‐stages and animals with cardiovascular disease obtained the least recommendations in the current study, even though these conditions are frequently associated with nutritional risk factors such as sarcopenia and cardiac cachexia, respectively (Freeman et al., 2003; Freeman, 2012; Gompf, 2005; Willems et al., 2017). Additionally, many respondents did not include supplementations of essential nutrients when recommending an HCD, raising the question whether long‐term use of an incomplete HCD is recognised as a nutritional risk factor. Eventually, failure to recognise nutritional risk factors can directly affect a patient's health and quality of life (Freeman et al., 2011), which is prevented by regular use of complete nutritional assessments (Bergler et al., 2016; Vandendriessche et al., 2017).

A lack of detailed nutritional knowledge and a feeling of incompetence in nutrition have been suggested to limit nutritional assessments and nutrition‐related discussions with animal caretakers during consultations (Bergler et al., 2016; Siebert et al., 2016). Indeed, many respondents indicated having insufficient knowledge about body composition assessment methods, which might signal a lack of general nutritional knowledge. Aforementioned can result in a reactive instead of a pro‐active attitude towards nutrition, as was found previously (Bergler et al., 2016). This attitude can have further consequences in owner compliance and adherence to nutritional recommendations, as it causes uncertainty in owners about the benefits of dietary change (Abood, 2008; MacMartin et al., 2018). Currently, compliance and adherence to dietary recommendations were estimated as being moderate, with costs as a common reason of non‐compliance. As owners willingly endure costs of veterinary care if they perceive that their animals’ well‐being or health is positively affected (Coe et al., 2007), a lack of concise and clear communication on the costs and the anticipated effects of nutritional recommendations may exist (Wayner & Heinke, 2006). Improving nutritional knowledge in veterinarians is therefore essential to increase confidence in nutrition‐related skills and communication and will result in an increased detection of nutritional risk factors and in improved owner compliance to nutritional recommendations.

The WSAVA guidelines can aid veterinarians in the process of incorporating nutrition in regular patient care, as they provide a clear overview of complete nutritional assessments and their use (Freeman et al., 2011). Since their release, the guidelines have been expanded with the ´Global Nutritional Toolkit’, which provides extensive supporting materials, such as videos on body composition assessment and dietary history forms (WSAVA, 2021). The nutritional assessment guidelines have been integrated in various nutrition‐related continuous education courses since their release, and are widely propagated via the WSAVA. Still, the majority of the respondents were unfamiliar with the guidelines, and similar findings were reported previously (Lumbis & de Scally, 2020). Thus, it is essential to increase awareness on the guidelines. This may be achieved by continued inclusion of nutritional assessment as a fifth vital assessment in continuing education, including non‐nutrition focused lectures and veterinary conferences, but also by expanding the role of nutrition in veterinary curricula (Becvarova et al., 2016; Lumbis & Scally, 2020). Another way the WSAVA may improve its reach is by collaboration with practice management software companies, to incorporate nutritional assessment as a (mandatory) field in patient records to increase the frequency of performed nutritional assessments.

There was a little difference between Belgian and Dutch respondents when considering the usage of nutritional assessments. Some differences were found in the nutritional recommendations for disease, and the means of follow‐up after recommending dietary change. These differences may be related to practice demographics, as Dutch practices tended to be larger and could allow for outsourcing of nutritional consultation to other employees. As such, Dutch veterinary nurses were responsible for approximately one‐fifth of all nutritional recommendations, suggesting lower involvement of Dutch veterinarians in nutrition. The influence of other factors, such as disease prevalence, differences in the years of experience of veterinarians in the sampled populations, an altered approach to nutrition in curricula (Becvarova et al., 2016) and attitudes of owners and veterinarians towards nutrition (Abood, 2008), cannot be excluded. Between‐country differences, however, were only moderately associated with the factor ‘country’, and it is to be expected that the cause of the differences is multi‐factorial. The current findings matched the results found previously in Germany (Bergler et al., 2016; Bruckner & Handl, 2020) and the United Kingdom (Lumbis & de Scally, 2020), and it is possible that the same factors apply for multiple countries, warranting further study.

A major limitation of the current study is the relatively small sample size, 222 Belgian and 201 Dutch respondents respectively, compared to the total amount of veterinarians in private practice, approximately 5700 in Belgium and 2400 in the Netherlands (FVE, 2015). Still, this sample size is comparable to previous similar studies (Bergler et al., 2016; Bruckner & Handl, 2020; Siebert et al., 2016). The demographics of the respondents were comparable to the general veterinary population of both countries, giving the possibility to extrapolate this data (FVE, 2015). The two‐year interval between the accessibility of the survey in Belgium and the Netherlands may also have affected the results, as progression in the knowledge of nutritional assessments might have been made in the intervening years.

Selection bias may have been introduced due to the methods of recruitment and the voluntary nature of the survey. The low completion rate of the survey by Dutch respondents when compared to the Belgian respond could reinforce this bias in comparisons made on later questions, as it is expected that motivated veterinarians tend to complete the survey. Additionally, estimation bias may be present, as the survey often required estimated information by the respondents without addressing the underlying motivations and context of these estimations. For instance, the percentage of HCDs in which a board‐certified nutritionist was involved was not determined, and the time for which these unbalanced diets were prescribed was not clear. Our findings may therefore differ from the actual situation. Finally, the current study did not address the specific factors that influence the use of nutritional consulting. As such, this may prove a direction for future studies in order to find specific solutions to increase the use of nutrition in every‐day practice.

In conclusion, the current data showed that nutritional assessment as described by the WSAVA nutritional assessment guideline was scarcely integrated in clinical practice. Most notably, nutritional evaluation by discussing diet and assessment of the animal's body composition were carried out irregularly, even though nutritional recommendations were often implemented in treatment plans. As a result, patients without clinical signs may be at risk of nutrition‐related disease due to underdiagnosed nutritional risks. General trends in the usage of nutritional assessments were similar in the examined countries, offering the possibility that the use of nutritional consulting is affected by the same factors in different countries. This offers the possibility to identify potential barriers to implement nutritional assessments in veterinary practices across countries. As veterinarians play a key role in identifying poor nutritional management or poor nutritional status, efforts to raise awareness on the importance of regular nutritional assessments should be continued. This includes additional education of students, but also extended post‐graduate trainings.

## CONFLICT OF INTEREST

Philippe Picavet is an employee of Hill's Pet Nutrition. Veerle Vandendriessche is an employee of Pavo. The other authors declare no conflict of interest.

## ETHICS STATEMENT

The authors confirm that the ethical policies of the journal, as noted on the journal's author guidelines page, have been adhered to. No ethical approval was required, as this study required no animal experiments.

## AUTHOR CONTRIBUTIONS

*Writing‐original draft, investigation, and data curation*: Niels R. Blees. *Conceptualisation, investigation, and writing‐review & editing*: Veerle L. Vandendriessche. *Methodology, supervision, and writing‐ review & editing*: Ronald J. Corbee. *Conceptualisation, visualisation, and writing‐ review & editing*: Phillipe Picavet. *Project administration, supervision, visualisation, and writing‐review & editing*: Myriam Hesta.

### PEER REVIEW

The peer review history for this article is available at https://publons.com/publon/10.1002/vms3.679.

## Data Availability

The data that support the findings of this study are available from the corresponding author upon reasonable request.

## 1

Survey containing questions about nutritional consulting in veterinary practice

**Table:**

		Questions	Answers
Composition of the practice	1	Do you treat dogs and cats in the practice?	Yes
			No
	2	What percentage of your patients are dogs or cats (on a yearly basis)?	More than 50%
			Less than 50%
	3	What percentage of all patients are dogs (on a yearly basis)?	Open answer
	4	What percentage of all patients are cats (on a yearly basis)?	Open answer
	5	What percentage of all patients are other small companion animals (birds, rodents, reptiles, etc.) on a yearly basis?	Open answer
	6	How many veterinarians work full‐time at the practice?	Open answer
	7	How many veterinarians work part‐time at the practice?	Open answer
	8	Please specify how many male and female veterinarians work at the practice.	
		Male	Open answer
		Female	Open answer
	9	How long have the veterinarians been graduated?	
		0–5 years	Open answer
		5–10 years	Open answer
		10–15 years	Open answer
		15–20 years	Open answer
		20–30 years	Open answer
		30–40 years	Open answer
		Longer than 40 years	Open answer
	10	Which specialisation?	
		Small animals	Open answer
		Horses	Open answer
		Production animals	Open answer
		Research	Open answer
		General	Open answer
	11	How many veterinarians are associates/co‐owner of the practice?	Open answer
	12	How many employees are supporting staff?	
	13	What function do these employees have?	
		Veterinary assistant	Open answer
		Accountancy	Open answer
		Maintenance	Open answer
		Other	Open answer
	14	Has any employee had specific training to answer questions concerning nutrition in the practice?	
			Yes
			No
	15	What education did this person receive?	
	16	Are additional courses concerning nutrition being attended?	
			Yes
			No
	17	If yes, what percentage of these courses is being attended by:	
		Veterinarians	Open answer
		Supporting staff	Open answer
Nutritional Screening	18	Where has the scale been placed?	
			Waiting room
			Consulting room
			Other
	19	Does the practice dispose of a specific cat scale?	
			Yes
			No
	20	Where is the cat scale located?	
			Open answer
	21	In what percentage of all cases is weight measured during each consultation?	
			Open answer
	22	In what percentage of all cases is weight written down in the patient's file?	
			Open answer
	23	Are changes in weight discussed with the owner?	
			Yes
			No
	24	Is the body condition score assessed during every consultation?	
			Yes
			No
	25	In what percentage of all cases is the body condition score assessed?	
			Open answer
	26	Is the BCS written down in the patient's file during each consultation?	
			Yes
			No
	27	In what percentage of all cases is the body condition score written down in the patient's file?	
			Open answer
	28	What scoring system is used in your practice?	
			Five‐point scale
			nine‐point scale
	29	What is the reason for not using the body condition score frequently?	
			Open answer
			
	30	Is the muscle condition score assessed during every consultation?	
			Yes
			No
	31	In what percentage of all cases is the muscle condition score assessed?	
			Open answer
	32	Is the muscle condition score written down in the patient's file during each consultation?	
			Yes
			No
	33	In what percentage of all cases is the muscle condition score written down in the patient's file?	
			Open answer
	34	What scoring system is used in your practice?	
			Description
			Scoring system
	35	What is the reason for not using the muscle condition score frequently?	
			Open answer
	36	Does your practice use nutritional surveys?	
			Yes
			No
	37	Who verifies the answers that were answered by the owner?	
			Veterinarian
			Assistant
	38	Is the information of the survey written down in the patient's file?	
			Yes
			No
	39	Who is responsible for writing down this information?	
			Open answer
	40	In case a nutritional survey is not used, in what percentage of all cases do you ask for:	
		Type of nutrition (brand, type, consistency)	Open answer
		Amount of food	Open answer
		Number of meals per day	Open answer
		Snacks (type, amount per day)	Open answer
		Housing of the animal	Open answer
		Activity of the animal	Open answer
	41	In what percentage of all cases is this information written down in the patient's file?	
		Type of nutrition (brand, type, consistency)	Open answer
		Amount of food	Open answer
		Number of meals per day	Open answer
		Snacks (type, amount per day)	Open answer
		Housing of the animal	Open answer
		Activity of the animal	Open answer
	42	Are the World Small Animal Veterinary Association (WSAVA) Nutritional Guidelines known to your practice?	
			Yes
			No
	43	What percentage of the veterinarians in your practice knows the WSAVA Nutritional Guidelines?	
			Open answer
The role of nutrition in the practice	44	What type(s) of nutrition does the practice sell?	
			Maintenance nutrition
			Therapeutic nutrition
			Raw nutrition
	45	Please specify what percentage of the total sales of nutritional sales is made by each category:	
			Maintenance nutrition
			Therapeutic nutrition
			Raw nutrition
	46	In how many of the following diseases in the cat are therapeutical nutrition recommended?	
		Lower urinary tract disease	Open answer
		Overweight	Open answer
		Diabetes mellitus	Open answer
		Orthopaedic disease	Open answer
		Kidney disease	Open answer
		Acute or chronic gastrointestinal disease	Open answer
		Dermal disease	Open answer
		Liver disease	Open answer
		Heart disease	Open answer
		Dental disease	Open answer
		Hyperthyroidism	Open answer
	47	In how many cases is specialised nutrition recommended for the following life phases in healthy cats?	
		Growth	Open answer
		Adult	Open answer
		Senior	Open answer
		Neutering	Open answer
	48	In how many of the following diseases in the dog are prescription diets recommended?	
		Overweight	Open answer
		Orthopaedic disease	Open answer
		Diabetes mellitus	Open answer
		Kidney disease	Open answer
		Heart disease	Open answer
		Acute or chronic gastrointestinal disease	Open answer
		Dermal disease	Open answer
		Lower urinary tract disease	Open answer
		Liver disease	Open answer
		Dental disease	Open answer
	49	In how many cases is specialised nutrition recommended for the following life phases in healthy dogs?	
		Growth	Open answer
		Adult	Open answer
		Senior	Open answer
		Neutering	Open answer
	50	What percentage of dietary changes is recommended by:	
		Veterinarian	Open answer
		Assistant	Open answer
	51	What percentage of owners applies the recommended diet?	
			Open answer
	52	In how many cases is the nutritional therapy applied until the recommended end?	
			Open answer
	53	Please specify the percentage of cases in which the following motivations are used to stop early with the diet:	
		Too expensive according to the owner	Open answer
		Not tasty enough according to the owner	Open answer
		No improvement of clinical condition according to the owner	Open answer
		Healed according to the owner	Open answer
		Other reason	Open answer
	54	Please specify any other reason that is being used by the owner to stop the diet:	
			Open
	55	In how many cases is a home‐made diet recommended as a (part of) a therapy?	
			Open
	56	When a home‐made diet is recommended, how do you choose the composition of this diet?	
			After consultation with a diplomate in nutrition
			Self‐acquired knowledge (book, internet, post‐graduate teaching, etc.)
	57	Without consultation with a diplomate in nutrition, in how many cases does the home‐made contain a:	
			Source of essential fatty acids
			Vitamin/mineral premix
	58	Please specify in how many cases, after a dietary change, the follow‐up is performed by:	
		A follow‐up consultation	Open
		Telephone	Open
		Written contact	Open
		Other	Open
	59	Who performs the follow‐up consultation?	
			Veterinarian
			Assistant
	60	In how many cases is the follow‐up written down in the patient's file?	
			Open
	61	What methods are used for extra motivation/to inform an owner about nutritional recommendations?	
		Gradual change to the new diet	
		Mentioning the amount of food per day	
		Mentioning the price per day	
		Mentioning the price per kilogram nutrition	
		Use of samples	
		Use of small packages	
	62	By choosing a specific brand/nutrition type, what criteria are of importance? Please order from high to low importance.	
		Evidenced‐based medicine	
		Innovations	
		Price	
		Taste	
		Partnership with the profession	
		Contact with a representative of the practice	
		Other	
	63	Which pet food brands does the practice sell?	
			Hill's
			Royal Canin
			Purina ProPlan
			Virbac
			Sanimed
			Specific
			Trovet
			Eukanuba/IAMS
			Other
			None
	64	Specific for neutered dogs and cats: how many owners are informed about:	
		The risk of the development of overweight	
		The necessity to reduce the amount of food or changing to a diet with a lower energy density	
	65	Specific for growing dogs and cats: how many owners are informed about:	
		Necessity to give nutrition that has been developed to support a growing animal	
		The correct time to switch to adult nutrition
		The risks of giving too many snacks during growth	
		The risks of giving supplements during growth	
	66	Which of the following information moments are organised by the practice?	
			Puppy and/or kitten parties
			Obesity clinics
			Behavioural clinics
			Senior screenings
			Other
			None
